# Correction: Knowledge, attitudes, and practices of organ, tissue, and cell donation in Nicaragua

**DOI:** 10.1371/journal.pgph.0004702

**Published:** 2025-05-20

**Authors:** The PLOS One Staff

## Notice of Republication

This article was republished on May 2, 2025, to correct an error in the title that was introduced during the typesetting process. The publisher apologizes for the error. Please download this article again to view the correct version. The originally published, uncorrected article and the republished, corrected articles are provided here for reference.

## Supporting information

S1 File Originally published, uncorrected article. (PDF)

S2 File Republished, corrected article. (PDF)

## References

[1] Navarrete J, Niño E, Moreno L, Bonilla IL, Gonzalez-Quiroz M. Knowledge, attitudes, and practices of organ, tissue, and cell donation in Nicaragua. PLOS Glob Public Health. 2025;5(3):e0004329. doi: 10.1371/journal.pgph.0004329 40100794 PMC11918347

