# Supplementary material for: Knowledge, attitudes, and practices of organ, tissue, and cell donation in Nicaragua
Source: PLOS Glob Public Health. 2025 Mar 18;5(3):e0004329. doi: 10.1371/journal.pgph.0004329 (PMC11918347; doi:10.1371/journal.pgph.0004329)
Supplement: S1 Table — (DOCX) [file pgph.0004329.s001.docx]

**S1 Table: Questions about knowledge of tissue and organ donation**

| **Questions** |  | **n** | **%** |
| --- | --- | --- | --- |
| What does organ and tissue donation mean to you? | Correct | 4,347 | 98,6 |
|  | Incorrect | 60 | 1.4 |
| What types of organ donors exist? | Correct | 3,621 | 82,2 |
|  | Incorrect | 786 | 17.8 |
| Do you know if organ donation and transplants are legal in Nicaragua? | Correct | 1,260 | 28,6 |
|  | Incorrect | 3,147 | 71.4 |
| Do you know if organ donation and transplants have been or are being performed in Nicaragua? | Correct | 1,344 | 30,5 |
|  | Incorrect | 3,063 | 69.5 |
| Have you heard about Law 847: Law on the Donation and Transplant of Organs, Tissues, and Cells for Human Beings? | Correct | 777 | 17,6 |
|  | Incorrect | 3,630 | 82.4 |
| Do you believe that any deceased person could become a donor? | Correct | 1,464 | 33,2 |
|  | Incorrect | 2,943 | 66.8 |
| Where are the places where organ transplants can be performed in Nicaragua? | Correct | 2,664 | 60,4 |
|  | Incorrect | 1,743 | 39.6 |
| What are the requirements to be an organ donor in Nicaragua? | Correct | 1,992 | 45,2 |
|  | Incorrect | 2,415 | 54.8 |
| Who do you think can donate organs? | Correct | 2,673 | 60,7 |
|  | Incorrect | 1,734 | 39.3 |
| Do you think donating an organ while alive could limit your quality of life? | Correct | 2,082 | 47,2 |
|  | Incorrect | 2,325 | 52.8 |
| What is the most transplanted organ in the country? | Correct | 1,554 | 35,3 |
|  | Incorrect | 2,853 | 64.7 |
| Which organs and tissues do you think can be donated? | Correct | 1,077 | 24,4 |
|  | Incorrect | 3,330 | 75.6 |
| How many lives can one person save by donating their organs? | Correct | 306 | 6,9 |
|  | Incorrect | 4,101 | 93.1 |
| **Total** |  | **4,407** | **100.0** |
