# Supplementary material for: Knowledge, attitudes, and practices of organ, tissue, and cell donation in Nicaragua
Source: PLOS Glob Public Health. 2025 Mar 18;5(3):e0004329. doi: 10.1371/journal.pgph.0004329 (PMC11918347; doi:10.1371/journal.pgph.0004329)
Supplement: S2 Table — (DOCX) [file pgph.0004329.s002.docx]

**Table S2: Questions about attitudes toward tissue and organ donation**

| **Items** | | **n** | **%** |
| --- | --- | --- | --- |
| I would not donate my organs if my religion prohibited it | Agree | 507 | 11.5 |
|  | Disagree | 3,900 | 88.5 |
| I would donate my organs if I were near death | Agree | 3,966 | 90.0 |
|  | Disagree | 441 | 10.0 |
| I think that a person who receives an organ transplant would improve their quality of life | Agree | 4,326 | 98.2 |
|  | Disagree | 81 | 1.8 |
| I believe that organ donation is an act of love for others | Agree | 4,314 | 97.9 |
|  | Disagree | 93 | 2.1 |
| I believe that people should not donate their organs because they belong to one person | Agree | 198 | 4.5 |
|  | Disagree | 4,209 | 95.5 |
| I believe that upon death, all people should automatically become organ donors | Agree | 1,266 | 28.7 |
|  | Disagree | 3,141 | 71.3 |
| I believe there should be a registry for people who wish to donate their organs | Agree | 4,188 | 95.0 |
|  | Disagree | 219 | 5.0 |
| I would only donate my organs if a close family member or friend who is very ill needed them | Agree | 1,302 | 29.5 |
|  | Disagree | 3,105 | 70.5 |
| I believe that only wealthy and famous people have better access and greater ease of receiving an organ transplant | Agree | 3,006 | 68.2 |
|  | Disagree | 1,401 | 31.8 |
| I believe that doctors might not do everything possible to save my life if I were an organ donor | Agree | 3,318 | 75.3 |
|  | Disagree | 1,089 | 24.7 |
| I believe that campaigns should be developed to educate and raise awareness within the population about organ donation and transplants | Agree | 4,257 | 96.6 |
|  | Disagree | 150 | 3.4 |
| I would be open to having a symbol added to my driver's license that identifies me as an organ donor | Agree | 3,933 | 89.2 |
|  | Disagree | 474 | 10.8 |
| I would feel fulfilled and happy to donate an organ and save someone's life | Agree | 4,236 | 96.1 |
|  | Disagree | 171 | 3.9 |
| Donating an organ would affect me emotionally and physically impact on you | Agree | 1,632 | 37.0 |
|  | Disagree | 2,775 | 63.0 |
| **Total** | | **4,407** | **100.0** |
