# Supplementary material for: Knowledge, attitudes, and practices of organ, tissue, and cell donation in Nicaragua
Source: PLOS Glob Public Health. 2025 Mar 18;5(3):e0004329. doi: 10.1371/journal.pgph.0004329 (PMC11918347; doi:10.1371/journal.pgph.0004329)
Supplement: S3 Table — (DOCX) [file pgph.0004329.s003.docx]

**Table S3: Questions about practices toward tissue and organ donation**

| **Questions** | | **n** | **%** |
| --- | --- | --- | --- |
| Have you ever donated blood? | Adequate | 2,133 | 48.4 |
|  | Inadequate | 2,274 | 51.6 |
| If you had a severely ill family member/friend in need of an organ transplant, would you be willing to donate an organ to them? | Adequate | 3,366 | 76.4 |
|  | Inadequate | 1,041 | 23.6 |
| Would you agree to sign a consent form authorizing the donation of your organs upon your death? | Adequate | 3,621 | 82.2 |
|  | Inadequate | 786 | 17.8 |
| If you were ill and required an organ transplant, would you be willing to receive a transplant of that organ? | Adequate | 4,308 | 97.8 |
|  | Inadequate | 99 | 2.2 |
| Which organs would you be willing to donate if the opportunity arose? | Adequate | 4,317 | 98.0 |
|  | Inadequate | 90 | 2.0 |
| If you wished to be an organ donor, according to your preferences, your organs would be allocated for: | Adequate | 4,404 | 99.9 |
|  | Inadequate | 3 | 0.1 |
| Have you shared your desire to donate your organs with any family member or friend? | Adequate | 1,221 | 27.7 |
|  | Inadequate | 3,186 | 72.3 |
| **Total** | | **4,407** | **100.0** |
