# Supplementary material for: Knowledge, attitudes, and practices of organ, tissue, and cell donation in Nicaragua
Source: PLOS Glob Public Health. 2025 Mar 18;5(3):e0004329. doi: 10.1371/journal.pgph.0004329 (PMC11918347; doi:10.1371/journal.pgph.0004329)
Supplement: S1 Questionnaire — (DOCX) [file pgph.0004329.s004.docx]

**Questionnaire: Knowledge, Attitudes, and Practices regarding organ, tissue, and cell donation in Nicaragua**

Doctors from the Medicine School at the National Autonomous University of Nicaragua (UNAN-León) have developed a survey on Knowledge, Attitudes, and Practices regarding organ, tissue, and cell donation. The aim is to assess the level of knowledge among individuals aged 18 and over residing in Nicaragua about organ donation. This will also help identify the attitudes and practices of the study population.

Your participation is entirely voluntary and anonymous. All the information you provide will be confidential. We kindly ask you to answer as sincerely as possible. We appreciate the time you are giving us in advance.

Your contributions are important, and by agreeing to participate in the study, you will be authorizing the researchers to use the provided information solely for academic research purposes.

I agree to participate in the study: ___Yes ___No (If “No,” end the survey).

Survey No.: ________

**I. General Information**

1. **Sex:**
   1. Male
   2. Female
2. **What is your current Age?** _____ years
3. **Place of residence:**
   1. Urban
   2. Rural
4. **What is your current Marital status?**
5. Single
6. Married
7. **What is your current Education level?**
8. Not study/Elementary school
9. High School/Technical
10. University/Professional
11. **What is your religion?**
12. Catholic
13. Evangelical
14. Other
15. None
16. **Do you work?**
    1. Yes
    2. No

II. Knowledge

Select only one option.

1. What does organ and tissue donation mean to you?

a. It is a voluntary and altruistic act in which a person donates organs and tissues to another person who needs them.

b. It is selling an organ for trafficking.

c. It is the obligation to give an organ to a person who needs it.

d. It is giving an organ to any person.

e. I don’t know.

1. What are the types of organ donors?

a. Living donors.

b. Deceased donors.

c. Both (living and deceased donors)

d. Persons with an illness

e. Persons who are about to die. f. I don’t know.

1. Do you know if organ donation and transplantation are legal in Nicaragua?

a. Yes.

b. No.

c. It used to be legal.

d. I don’t know.

1. Do you know if organ donation and transplantation have been or are being practiced in Nicaragua?

a. Yes.

b. No.

c. It used to be practiced.

d. I don’t know.

1. Have you heard about Law 847: Law on the Donation and Transplantation of Organs, Tissues, and Cells for Humans being?

a. Yes.

b. No.

c. I don’t remember.

1. Do you believe that any deceased person could become a donor?

a. Yes.

b. No.

c. Only persons who did not die from illness.

d. Only persons who died in a traffic accident. e. I don’t know.

1. What are the places where organ transplants can be performed in Nicaragua? Select one or more options:
2. Public hospitals
3. Private hospitals or clinics
4. Hospitals attended by foreign medical brigades
5. Hospitals or clinics specialized in transplants
6. I don’t know.
7. What are the requirements to be an organ donor in Nicaragua? Select one or more options:
   1. Being in good health and of legal age.
   2. Being deceased.
   3. Having compatibility with the person to whom the organ will be donated.
   4. None, no requirement is necessary.
   5. Having a demonstrable family or emotional bond with the person to whom the organ will be donated.
   6. At least 2 close family members agreeing with the donation.
   7. Not having children.
   8. Being of sound mind.
8. Who do you think can donate organs?

a. Children.

b. Youths.

c. Adults.

d. Elderly.

e. Everyone.

1. Do you think donating an organ while alive could limit your quality of life?

a. Yes.

b. No.

c. Partially.

d. I don’t know.

1. What is the most transplanted organ in the country?

a. Lung.

b. Kidney.

c. Heart.

d. Corneas.

e. Liver.

f. I don’t know.

1. Which organs and tissues do you think can be donated? Select one or more options:
   1. Lungs
   2. Kidneys
   3. Liver
   4. Corneas
   5. Heart
   6. Intestine
   7. Bone
   8. Skin
   9. Pancreas
   10. Bone marrow
   11. All
2. How many lives can one person save by donating their organs?

a. Can save only 1 life

b. Can save the lives of 3 people.

c. Can save the lives of 5 people.

d. Can save the lives of 8 people.

e. Can save the lives of 10 people.

f. I don’t know

**III. Attitudes**

Select only one option.

1. **I would not donate my organs if my religion prohibited it**

1. Agree
2. Disagree

2. **I would donate my organs if I were near death**

1. Agree
2. Disagree

3. **I think that a person who receives an organ transplant would improve their quality of life**

1. Agree
2. Disagree

4. **I believe that organ donation is an act of love for others**

1. Agree
2. Disagree

5. **I believe that people should not donate their organs because they belong to one person only**

1. Agree
2. Disagree

6. **I believe that upon death, all people should automatically become organ donors**

1. Agree
2. Disagree

7. **I believe there should be a registry for people who wish to donate their organs**

1. Agree
2. Disagree

8. **I would only donate my organs if a close family member or friend who is very sick needed them**

1. Agree
2. Disagree

9. **I believe that only wealthy and famous people have better access and greater ease in receiving an organ transplant**

1. Agree
2. Disagree

10. **I believe that doctors might not do everything possible to save my life if I were an organ donor**

1. Agree
2. Disagree

11. **I believe that campaigns should be developed to educate and raise awareness within the population about organ donation and transplants**

1. Agree
2. Disagree

12. **I would be open to having a symbol added to my driver's license that identifies me as an organ donor**

1. Agree
2. Disagree

13. **I would feel fulfilled and happy to donate an organ and save someone's life**

1. Agree
2. Disagree

14. **Donating an organ would have an emotional and physical impact on you**

1. Agree
2. Disagree

**IV. Practices**

Select only one option.

1. **Have you ever donated blood?**

a. Yes

b. No

2. **If you had a severely ill family member or friend in need of an organ transplant, would you be willing to donate an organ to them?**

a. Yes

b. No

c. I don’t know

3. **Would you agree to sign a consent form authorizing the donation of your organs upon your death?**

a. Yes

b. No

c. I don’t know

d. Let my family decide

4. **If you were ill and required an organ transplant, would you be willing to receive a transplant of that organ?**

a. Yes

b. No

5. **Which organs would you be willing to donate if the opportunity arose? Select one or more options:**

1. Lungs
2. Kidneys
3. Corneas
4. Liver
5. Heart
6. Intestines
7. Bones
8. Skin
9. Pancreas
10. Bone marrow
11. All
12. None

6. **If you wished to be an organ donor, according to your preferences, your organs would be allocated for:? Select one or more options:**

1. Friend
2. Family member
3. Acquaintance
4. Any person

7. **Have you shared your desire to donate your organs with any family members or friends?**

a. Yes

b. No

Thank you for your participation
