# Supplementary material for: Knowledge, attitudes, and practices of organ, tissue, and cell donation in Nicaragua
Source: PLOS Glob Public Health. 2025 Mar 18;5(3):e0004329. doi: 10.1371/journal.pgph.0004329 (PMC11918347; doi:10.1371/journal.pgph.0004329)
Supplement: Attachment — Submitted filename: Rebuttal letter.docx [file pgph.0004329.s009.docx]

**W. Alton Russell, PhD**

**Academic Editor**

**PLOS Global Public Health**

January 3, 2025

Dear Dr Alton Russell,

**Re: Knowledge, Attitudes, and Practices of Organ, Tissue, and Cell Donation in Nicaragua. Manuscript ID: PGPH-D-24-02008**

Thank you for your interest in our paper and for providing us with the opportunity to address the reviewers' and editor's comments. We have made the suggested revisions to the manuscript and have outlined our responses to the points raised on the following page.

We look forward to hearing from you again.

Yours sincerely,

Jasley Navarrete, MD

Corresponding author

**Point by point response to referees comments**

| ***Referee*** | ***Comments from reviewer*** | ***Response to comments*** |
| --- | --- | --- |
| *Editor Comments* | *First, the manuscript would be more informative for international audiences if your approach, your findings, and the Nicaraguan context were better situated within the international context.* | *We have addressed the reviewer comments by providing more details in the method section and editing the discussion.* |
|  | *Second, the level of evidence available to justify the validity of your survey and its reliability for the population who responded could be better characterized.* | *We have calculated the coefficient of Cronbach’s alpha* |
| *Reviewer 1* | *The abstract presents the information correctly.* | *We appreciate your positive comment* |
|  | *The introductory part is considered adequate and consistent with the objectives and supporting literature.* | *We appreciate your positive comment* |
|  | *Methodology. This section could be developed. For example, it would be interesting to know the procedure. As for the evaluation instrument used, it is not a validated or standardized questionnaire. These instruments are currently being validated, for example PCID-DTO-RIOS. The instrument used for this study does not describe the psychometric characteristics of the instrument, for example, reliability and validity.* | *We appreciate the thoughtful remark made by the reviewer. We have provided my details in the study design and population, sampling, and questionnaire content and data collection.* |
|  | *The description of the results are clearly stated and coherent with the proposed objectives.* | *We appreciate your positive comment* |
|  | *Regarding the discussion and conclusions, perhaps the results obtained could be compared, to a greater extent, with the results of other similar studies carried out in other countries. For example, there are many studies carried out in Spain, as it is a leading donation country. This would be an enrichment in terms of the quality of the work presented. In general, it is a correct work, although the different sections could be completed to increase its quality.* | *We have made substantial changes in the discussion, adding Nicaraguan contextual information and comparing the results with data from India, Spain, etc.* |
| *Reviewer 2* | *The sample bias- mostly women, younger, educated etc…most eligible deceased donors are older than this sample and likely not represent the general population…it feels like a bit more needs to be said and done to address the actual make-up of the population to test these further.* | *Thank you for raising this important comment. We have provided the following information:*  *“We notice that there is also a notable gender disparity in participation rates, with more females engaging in organ donation-related discussions and research. This aligns with global trends observed in other countries, where women are often more receptive to healthcare initiatives. In the Nicaraguan context, cultural factors, such as women’s greater involvement in healthcare decision-making within families and communities, contribute to their higher participation rates. Additionally, women tend to be more active in seeking health-related information, making them more likely to support and participate in organ donation initiatives. In contrast, men, especially in rural areas, may be less involved in healthcare discussions, which could explain the lower participation rates among them”* |
|  | *There is nothing here about live donation. Was this explored?* | *In Nicaragua, there is no organ donation program for deceased donors; only transplants from living donors with first-degree consanguinity are performed in private healthcare services* |
|  | *Limited Contextualisation: While the results are well-detailed, the discussion could benefit from more contextual comparison with similar studies in other low- and middle-income countries or regions facing similar challenges, and put more into context with this population – i.e. the recommendations are very generic and there is no priority given or suggestions how things can be implemented in a lower-middle country which has highly limited resources? I do not disagree with the discussion but it is very generic – what is specific to this population and demographic – or is it that the quick wins seen in developed countries and services are what need to be adapted – if so what is the priority and how can we make room for these in a country with limited resources/funds.* | *Thank you for raising this important comment. We have provided local context and compared the results with similar studies conducted in LMICs.* |
|  | *The law while interesting comes out of the blude and it is not clear what the question or use of it is trying to achieve, i.e. it does not explore in depth how this law has been implemented or its impact on public attitudes and behaviours, much more needs to be made of this if it is to be included, when introduced, what was there before, how was it advertised, does it work, what is the evidence etc etc…is it having impact on trust positive/negative etc etc* | *Law 847 – “Law on the Donation and Transplantation of Organs, Tissues, and Cells in Nicaragua regulates both the donation and transplantation of organs, tissues, and cells from living and deceased individuals” was introduced in 2013, representing a significant step toward regulating organ donation and transplantation in Nicaragua. Prior to this law, the country lacked comprehensive legal frameworks to govern organ donation, leading to inconsistent practices and limited public awareness. The survey highlighted a significant knowledge gap regarding the Law 847, stemming from insufficient public dissemination or the absence of an educational program to raise awareness among the population. Additionally, the country lacks an operational organ transplant program for both living or deceased donors. However, the survey also revealed that individuals with higher education levels tend to have greater knowledge and more likely to seek out information on the benefits and procedures, resulting in more positive attitudes toward it.20 This trend is particularly evident in urban areas, where access to educational resources is more readily available. Similar findings have been reported in southern India,24 Nigiria,25,26 Paskitan,27 Egypt28 and Spain21 where participants with higher education demonstrated better knowledge of the concept of organ donation and transplantation, consent processes, and the ethical issues surrounding organ donation. Studies suggest that targeted educational interventions could help bridge the knowledge gap, particularly among populations with lower educational levels. In some LMICs, awareness campaigns aimed at students, healthcare professionals, and community leaders have been successful in improving knowledge and attitudes about organ donation.* |
|  | *I was surprised to see that many of the questions appear to have open free text responses – and are omitted? If this is the case, why so? Much more could be done in the analysis to see what people are actually saying and where their KAPs might be amenable to intervention to strengthen this work and suggest more specific strategies for this specific population.* | *Thank you for raising this important comment. We added the following text in questionnaire section:*  *“A structured questionnaire with closed-ended questions was developed through a comprehensive multi-step process. The initial drafted was self-designed, drawing from existing, validated questionnaires recognized for their high reliability and validity in the context of organ and kidney transplantation.15-17 This draft was then adapted to the Nicaraguan population with inputs from three experts in epidemiology, health statistics, and psychology”* |
|  | *I am unable to comment in detail on the statistics, but as alluded to, it feels like more nuanced work is needed to address the KAP needs – this is rather a starting point than an end.* | *We appreciate your comment* |
|  | *It is very interesting to read about the numbers and disparities and scarcity of donation – and that males are mostly getting kidneys – women are overrepresented on the waitlist for kidneys in the UK.* | *There is an epidemic of Chronic Kidney Disease of unknown etiology (CKDu) in Mesoamerica, with Nicaragua being the second country in the region with the highest mortality rate. This disease predominantly affects disadvantaged rural agricultural workers, mostly men aged 20-40 years. A small proportion of patients with end-stage renal disease (ESRD) have the opportunity to receive hemodialysis (HD) due to the limited capacity of the healthcare system. Furthermore, there is no active organ transplant program in the country, either in the public or private sector, and there is no waitlist for kidney transplants.* |
|  | *Some of the questions may be too general or simplistic to gauge nuanced understanding (e.g., “What is organ and tissue donation?”), as is suggested in the first line of the discussion – especially since none of the free text responses were analyzed.* | *Thank you for highlighting this issue. I fully agree with your comment. We decided to use general closed-ended questions because we needed to introduce the topic to the participant, and we were unsure of their level of education on organ donation. We also recognize the limitation that we did not collect open-ended responses, which could have complemented the analysis and provided a deeper understanding of the participants' perspectives* |
|  | *"What are the requirements to be an organ donor in Nicaragua?" this would require specialist knowledge not known by any general public.* | *According to the Law 847: Law on the Donation and Transplantation of Organs, Tissues, and Cells in Nicaragua regulates both the donation and transplantation of organs, tissues, and cells from living and deceased individuals. The requirements for being an organ donor in Nicaragua are as follows:*  ***Living donors***   1. *Legal and mental capacity: The donor must be of legal age and in full possession of their mental faculties.* 2. *Adequate health status: The donor must have an appropriate state of health to undergo organ extraction.* 3. *Informed consent: The donor must be informed about the risks of donation, possible complications, foreseeable outcomes, and resulting limitations. Consent must be provided through the informed consent process, as outlined by the Ministry of Health regulations.* 4. *Relationship with the recipient: The donor must have a marital relationship or a demonstrated stable de facto union with the recipient.* 5. *Compliance with protocols: The donor must meet all established medical and legal protocols.* 6. *Compatibility: The donor must have blood type compatibility and histocompatibility with the recipient, based on medical testing in accordance with Ministry of Health regulations.* 7. *Safety considerations: Organ removal (such as one organ of a paired set) must not pose a foreseeable risk to the donor’s health.* 8. *Clinical guidelines: The donation process must adhere to clinical practice guidelines established by the Ministry of Health.* 9. *Other conditions: Any additional conditions deemed necessary by the Ministry of Health must be met, as specified in clinical guidelines.*   *Deceased donation:*   1. *Donation can proceed if the individual expressed their desire to donate during their lifetime, either through official documents or registers approved by the Ministry of Health.* 2. *In the absence of expressed consent, authorization can be provided by immediate family members, following the order of kinship established by the law:*    A) *The spouse or the person who lived with the deceased in a stable de facto union for no less than three years, continuously and uninterruptedly.*    B) *Sons or daughters over the age of 21.*    C) *Parents*    D) *Siblings over the age of 21.*    E) *Grandchildren over the age of 21*    F) *Grandparents*    G) *Blood relatives over the age of 21, up to the fourth degree of consanguinity*    H) *Relatives by marriage (affinity) over the age of 21, up to the second degree of affinity.* |
|  | *The two-step Likert scale (Agree/Disagree) restricts nuanced responses, many people are indifferent, confused, uncertain when it comes to organ donation.* | *We appreciate the reviewer’s comment and agree with the suggestion. During the questionnaire validation process, we consulted with a psychology expert specializing in the evaluation of knowledge, attitudes, and practices. Based on their advice, we decided to adopt a two-step Likert scale. The expert recommended excluding a neutral or indifferent option, as individuals often default to these options, which could reduce the interpretive value and clarity of the responses* |
|  | *While the questionnaire asks about willingness and consent, it does not explore the reasons behind the lack of action (e.g., "Why have you not communicated your desire to donate?").* | *Thank you for pointing this out. We agree that understanding the reasons behind the lack of action, such as why individuals have not communicated their desire to donate, would provide valuable insights. While this aspect was not included in the current version of the questionnaire, we recognize its importance and will consider incorporating such questions in future studies or follow-up surveys. This addition could help identify barriers to communication, whether they stem from cultural, social, or personal factors, and provide a more comprehensive understanding of the topic* |
|  | *Questions also assume an understanding of concepts like blood donation, which may not be universal or uniformly accessible in Nicaragua, at the same time a recommendation might be to increase blood donors thus leading to tailored comms about organ donation.* | *Thank you for raising this important point. Blood donation campaigns are often more familiar and visible in many communities, making them a valuable platform for initiating tailored communication strategies about organ donation. By leveraging the existing awareness and engagement around blood donation, local authorities could develop targeted outreach initiatives to educate the public and promote understanding and acceptance of organ donation. This integrated approach could bridge knowledge gaps while simultaneously encouraging greater participation in both blood and organ donation programs* |
| *Formatting amendments* | *Questionnaire* | *Attached as supplementary material* |
|  | *Data sharing statement* | *Please contact the relevant authors for data requests or any additional information* |
